# *Theileria parva*: a parasite of African buffalo, which has adapted to infect and undergo transmission in cattle

**DOI:** 10.1016/j.ijpara.2019.12.006

**Published:** 2020-05

**Authors:** W Ivan Morrison, Johanneke D. Hemmink, Philip G. Toye

**Affiliations:** a The Roslin Institute, Royal (Dick) School of Veterinary Studies, University of Edinburgh, Easter Bush Campus, Roslin, Midlothian EH25 9RG, UK; b The International Livestock Research Institute, PO Box 30709, Nairobi, Kenya

**Keywords:** *Theileria parva*, Cattle, African buffalo, Genetic diversity, Tick transmission

## Abstract

The tick-borne protozoan parasite *Theileria parva* causes an acute, often fatal disease in cattle throughout a large part of eastern and southern Africa. Infection of African buffalo (*Syncerus caffer*) is also widespread in this region but does not cause clinical disease in this species. This difference most likely reflects the evolutionary history of the parasites in these species, in that cattle were only introduced into Africa within the last 8000 years. In both hosts, *T. parva* establishes a carrier state, involving persistence of small numbers of parasites for many months following the acute phase of infection. This persistence is considered important for maintaining the parasite populations. Although cattle and buffalo parasites both produce severe disease when transmitted to cattle, the buffalo-derived parasites are usually not transmissible from infected cattle. Recent studies of the molecular and antigenic composition of *T. parva*, in addition to demonstrating heterogeneity in the populations in both host species, have revealed that infections in individual animals are genotypically mixed. The results of these studies have also shown that buffalo *T. parva* exhibit much greater genotypic diversity than the cattle population and indicate that cattle parasites represent a subpopulation of *T. parva* that has adapted to maintenance in cattle. The parasites in cattle and buffalo appear to be maintained largely as separate populations. This insight into the genotypic composition of *T. parva* populations has raised important questions on how host adaptation of the parasite has evolved and whether there is scope for further adaptation of buffalo-maintained populations to cattle.

## Highlights

• *Theileria parva* parasites show extensive genotypic diversity and undergo frequent genetic recombination during tick transmission.
• *Theileria parva* maintained in cattle is much less genotypically diverse than the buffalo-maintained population.
• *Theileria parva* transmitted from buffalo to cattle usually fails to differentiate to the tick-transmissible stages in cattle.
• These differences have resulted in the parasites in the two hosts being maintained largely as separate populations.

## Graphical abstract

**Figure:**
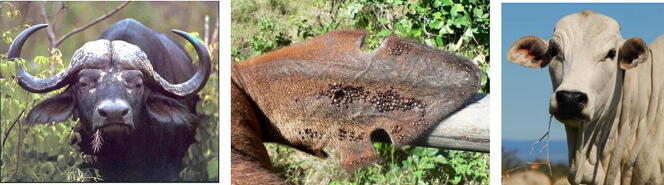

## 1 Introduction

The *Theileria* genus includes a large number of species of protozoan parasites that infect wild and domestic ruminants in tropical and subtropical regions of the world (Uilenberg, 1981, Morrison, 2015). They are tick-borne organisms and the geographical distribution of each species is determined largely by the distribution of the respective tick vector species. *Theileria* undergo sequential development in leukocytes and erythrocytes (schizont and piroplasm stages, respectively) in their mammalian hosts, with piroplasms being the stages infective for the tick vectors. Some species multiply predominantly in erythrocytes whereas others multiply mainly in leukocytes. There is wide variation in pathogenicity among the species that infect domestic ruminants, some being relatively benign whereas others cause severe clinical disease. The latter include the two most important species affecting cattle, *Theileria parva* and *Theileria annulata,* which cause high levels of morbidity and mortality in susceptible stock (Uilenberg, 1981, Irvin and Morrison, 1987). Replication of these parasites involves induction of proliferation of the infected host leukocytes and synchronous division of the parasite with the host cell, resulting in rapid clonal expansion of the infected cells (Hulliger et al., 1964, Dobbelaere and Rottenberg, 2003, von Schubert et al., 2010). This intimate host-parasite relationship enables parasitized cells to be cultured in vitro as continuously growing cell lines (Brown et al., 1973). *Theileria annulata* is transmitted by a number of species of *Hyalomma* ticks and is found in a large subtropical zone extending from southern Europe and North Africa through the Middle East into Asia (Pipano, 1989), whereas *T. parva,* transmitted most commonly by *Rhipicephalus appendiculatus*, is present throughout a large part of eastern and southern Africa (Irvin and Morrison, 1987, Lawrence et al., 1994a). These parasites also infect the Asian and African species of buffalo (*Bubalis bubalis* and *Syncerus caffer,* respectively). Infection of African buffalo with *T. parva* occurs throughout the region where the tick vector occurs, but in contrast to cattle, buffalo do not appear to suffer disease. However, they represent a reservoir of infection for transmission of the parasite to cattle and hence are an important consideration in the application of disease control measures.

For most of the 20th century, there was debate as to whether *T. parva* found in cattle and buffalo are separate species or sub-species. This distinction was based largely on observed differences in the characteristics of the diseases produced following transmission to cattle and the tick transmissibility of the parasites. However, subsequent findings indicating genetic and antigenic similarity of the parasites led to the view that they do indeed represent a single species. Recent studies of *T. parva* populations in cattle and buffalo have highlighted the extent of antigenic and genotypic diversity of the parasites in both host species and confirmed the basis of antigenic cross-reactivity between the populations. However, buffalo parasites were found to have much greater diversity than those in cattle, supporting previous suggestions that the cattle parasites represent a subset of the overall *T. parva* population. Herein, we will briefly review these recent advances, together with relevant historical data, and consider how evolution of the parasite has shaped the genetic composition and host preference of the *T. parva* populations currently found in cattle and buffalo.

## 2 Cattle are recent immigrants into Africa

Domestic cattle originated from a wild progenitor, the Auroch (*Bos primigenius*), which was widely distributed in Asia and Europe but eventually became extinct in the early 18th century. Archaeological records have indicated that domestication of cattle occurred in the Middle East and Asia, and that migration of humans with their animals westwards and through the horn of Africa resulted in the introduction of cattle into Europe and Africa (Hanotte et al., 2002, Gifford-Gonzalez and Hanotte, 2011). Several waves of migration into Africa occurred between 5000 and 10,000 years ago, the descendants of which include the indigenous, predominantly *Bos indicus* and *B. indicus*/*Bos taurus* cross-breeds currently present in *Theileria*-endemic regions in Africa. Much more recently, European breeds used for intensive production have been introduced into Africa at various times over the last 150 years. Hence, since *T. parva* is confined to Africa, cattle have had a relatively short period of time for evolutionary adaptation to the parasite. Consequently, European breeds and many improved *B. indicus* African breeds suffer severe disease and high levels of mortality when exposed to *T. parva* challenge. However, some indigenous East African *B. indicus* breeds, which have been maintained in the presence of *T. parva* challenge, show a degree of resistance to the disease, but still suffer mortality rates of up to 20% (Barnett, 1957, Stobbs, 1966, Ndungu et al., 2005, Kiara et al., 2014).

## 3 Key historical findings

### 3.1 Cattle-maintained *T. parva*

Early studies of theileriosis in Africa, including the discovery of *T. parva,* are discussed in detail elsewhere (Lawrence, 1992, Norval et al., 1992) and will only be summarised briefly here. Detailed investigation of an acute, rapidly fatal disease in cattle, which first appeared in southern Rhodesia (currently Zimbabwe) in 1902 and rapidly spread locally and southward into South Africa, led to the identification of the causal agent as an intracellular protozoan parasite transmitted by the three-host tick, *R. appendiculatus* (Theiler, 1904, Lounsbury, 1904). The parasite was subsequently named *T. parva* and the disease referred to as East Coast fever (ECF). Much more recent studies showed that another closely related tick species, *Rhipicephalus zambesiensis*, found only is southern Africa, also acts as a vector and it has been reported to be a more efficient vector than *R. appendiculatus* (Lawrence et al., 1983, Blouin and Stoltsz, 1989). The origin of the disease outbreak in 1902 was attributed to importation of cattle from Tanzania, prompted by severe depletion of cattle in southern Africa in the previous 10 years, caused primarily by the rinderpest pandemic that had swept through the continent in the 1890s and by the demands of the recent Boer war (Reviewed by Lawrence, 1992). Prior to its appearance in southern Africa, the disease had not been formally identified in East Africa, but several reports of similar disease appeared in the following years (discussed in Norval et al., 1992). In 1912, Montgomery (Anon, 1912) showed that recovered animals in Kenya were immune to challenge with ticks infected with the South African parasite (provided by Theiler), indicating identity of the causal agents.

Elucidation of the aetiology and transmission of the disease in South Africa led to implementation of a series of control measures aimed at eradicating the disease, which included regular dipping of cattle to prevent tick infestation, intensive disease surveillance, slaughter of infected animals following an outbreak and quarantine of affected farms (reviewed by Lawrence, 1992). Maintenance of this control programme over several decades eventually resulted in the disappearance of ECF from South Africa by the mid-1950s.

For many decades cattle that recovered from ECF were considered to have developed a “sterile immunity” (Neitz, 1957, Barnett, 1968), based largely on the inability to detect infected erythrocytes microscopically. However, in 1986 experiments involving tick feeding on cattle that recovered following treatment with the theilericidal compound buparvaquone demonstrated transmission of infection (Dolan, 1986). The development of persistent infection, referred to as the carrier state, following recovery from natural infection was then confirmed by successful transmission with ticks fed on the animals (Young et al., 1986). Transmission of infection was observed up to 13 months after the cattle had been removed from field challenge, clearly demonstrating that infection with *T. parva* persists well beyond the acute phase of infection. This carrier state is now readily detected using PCR assays (Skilton et al., 2002). Mathematical modelling of transmission dynamics has indicated that the carrier phase of infection (compared with the acute phase) accounts for a large proportion of the parasite infections in field tick populations in ECF-endemic areas (Medley et al., 1993).

### 3.2 Buffalo-derived *T. parva*

An acute disease in cattle caused by a morphologically similar parasite, but exhibiting epidemiological differences from ECF, was recognised in Zimbabwe in 1934 (Lawrence, 1935, Lawrence, 1936). Although the disease showed some features in common with ECF, including acute onset and a high level of mortality, affected animals had lower levels of schizont-infected leukocytes in lymphoid tissues and no, or very few, detectable piroplasms in erythrocytes. The disease appeared to be associated with contact with buffalo. It was noted that when groups of cattle containing animals that had recovered from the disease were removed from buffalo contact to tick-infested pasture, no further cases of the disease occurred, inferring that the animals did not represent a source of infection for ticks. The same disease was reported in South Africa in the 1950s (Neitz, 1955) and shown to be transmitted to cattle by *R. appendiculatus* ticks that had fed on buffalo (Neitz et al., 1955). The parasite was named *Theileria lawrencei* and the disease was termed Corridor Disease, referring to the local region in South Africa where it was initially recognised. Earlier observations indicating lack transmission from infected cattle were confirmed by experiments showing that ticks fed on recovered cattle did not transmit the infection (Neitz, 1955). Corridor disease continued to occur in South Africa after the eradication of ECF in the 1950s.

A similar disease in cattle caused by *Theileria lawrencei*, also arising from transmission by ticks from buffalo, was subsequently recognised in East Africa (Brocklesby and Barnett, 1966). It is now widely accepted that most buffalo residing in *R. appendiculatus*-infested areas are infected with this parasite (Young et al., 1978). Tick transmission from infected buffalo to buffalo and cattle in Kenya has been demonstrated consistently (Young et al., 1973, Young et al., 1977b) and, in one study using a single experimentally infected buffalo held in tick-free accommodation, tick transmission was detected up to 880 days after infection (Grootenhuis et al., 1987).

Despite the observed differences between *T. parva* and *T. lawrencei*, subsequent findings revealed antigenic similarities and experiments, involving immunisation and challenge of cattle, demonstrated cross-protection between some combinations of *T. parva* and *T. lawrencei* isolates (eg., Radley et al., 1979; discussed in Section 7 below). Moreover, repeated tick passage of certain East African *T. lawrencei* isolates through cattle was reported to result in a gradual change in the behaviour of the parasite to eventually resemble *T. parva* (Barnett and Brocklesby, 1966a, Young and Purnell, 1973, Maritim et al., 1992) (discussed further in Section 6). Consequently, *T. lawrencei* was re-classified as *T. parva*, but the parasites were still considered to be sub-types, referred to as *T. parva parva* and *T. parva lawrencei* (Uilenberg, 1981). With further accumulating evidence of antigenic and molecular similarities, support for this subspecies classification waned, and more recently the parasites have merely been referred to as *T. parva* of cattle or buffalo origin.

## 4 Tick transmission

Transmission of *T. parva* by the tick vector occurs trans-stadially, ie. parasites ingested by larval and nymphal stages during feeding on an infected animal are transmitted to a new host by nymphal and adult ticks, respectively. The parasite is haploid throughout development in the mammalian host, but undergoes a complex cycle of development in the tick (Norval et al., 1992, Mehlhorn et al., 1994), during which there is a transient diploid phase. Piroplasms ingested by the tick differentiate to gametes, which fuse to form zygotes, and further development in the tick salivary glands includes meiotic division, yielding (haploid) mammal-infective sporozoites. This process facilitates sexual recombination between different parasite strains in the tick (discussed in Section 8.3).

Transmission in the field is strongly influenced by climatic conditions. In East Africa, transmission can occur more or less throughout the year, although the activity and abundance of ticks usually show seasonal variation influenced by temperature and rainfall. By contrast, there is a pronounced seasonal pattern of tick transmission in southern Africa, which is due to the unfed adult *Rhipicephalus* ticks entering an inactive or quiescent phase, known as diapause, during the “winter” season (Norval et al., 1991). This results in a degree of synchronisation of the tick life cycle and restricts life cycle development of most ticks to completion of one generation per year (Norval et al., 1991). This pattern of tick development places certain constraints on transmission of *T. parva*, most obviously a requirement for the parasite to persist in the mammalian host during the winter period to ensure survival and maintenance of the parasite population.

## 5 Transmission between buffalo and cattle

The capacity to undergo tick transmission is a key factor that distinguishes infections in cattle with cattle-maintained and buffalo-derived *T. parva*. Both populations of parasites are efficiently transmitted by ticks in their respective host species and both establish a carrier state, enabling transmission to occur from animals harbouring low levels of parasites. The efficiency of this transmission is particularly apparent in carrier cattle, which usually do not have microscopically detectable parasites in their blood. Since the piroplasm stage of *T. parva* undergoes little, if any, replication and erythrocytes have a short lifespan, the establishment of a carrier state also depends on persistence of small numbers of infected leukocytes, which provide a continual source of merozoites for infection of erythrocytes.

By contrast to the efficient transmission of parasites from infected buffalo, ticks fed on cattle infected with the same buffalo-derived parasites usually fail to transmit infection. This is associated with detection of no, or occasionally very low, levels of piroplasms in cattle during the acute phase of infection with buffalo-derived parasites. In a recent study, which monitored a group of 24 cattle introduced into an area grazed by buffalo, all animals developed typical corridor disease, but only two had very low levels of detectable piroplasms (Sitt et al., 2015). The observed variation in duration of clinical disease in these animals argues against the suggestion that cattle infected with buffalo-derived parasites may die before piroplasms have a chance to develop. The paucity of piroplasms and lack of transmission of these parasites by ticks appears to be due to a failure of schizonts in infected lymphocytes to undergo merogony, since microschizonts (ie. schizonts undergoing merogony) are rarely detected in lymphoid tissues (Young et al., 1977b). Hence, there is a failure to generate the erythrocyte-infective stages.

## 6 Behavioural “transformation” of buffalo *T. parva* in cattle

While numerous attempts have failed to transmit buffalo-derived parasites from cattle, instances of transmission of such parasites have been reported in East Africa and serial tick transmission was found to result in altered behaviour of the parasites to resemble that of cattle-maintained *T. parva*. This so-called “transformation” was first reported by Barnett and Brocklesby (1966a), who infested susceptible cattle with ticks that had fed on a *Theileria*-infected buffalo, and then conducted tick passages to further naïve cattle. Although the first and second passages usually did not result in microscopically detectable parasites, transmission was successful in a proportion of the tick feeds. Further tick passages resulted in progressively increasing levels of parasites in both erythrocytes and lymphocytes and severe, fatal disease in some animals, which by the fifth and sixth passages resembled disease produced by cattle-maintained *T. parva*. Unfortunately, methods to cryopreserve the parasites were not available at that time and therefore further studies on this parasite are not possible. In 1973, Young and Purnell also reported successful tick transmission of a buffalo isolate of *T. parva* and observed progressively increasing levels of piroplasm parasitaemia upon serial tick passage. This cattle-adapted parasite, *T. parva* (Serengeti), was subsequently used as one of the components of a live vaccine against *T. parva* (discussed below). Recent studies of the component parasites of the current Muguga cocktail vaccine revealed that the satellite DNA profile and the genome sequence of *T. parva* (Serengeti) showed a high level of identity to that of the cattle isolate *T. parva* (Muguga) (Norling et al., 2015, Hemmink et al., 2016). The level of identity indicated that *T. parva* (Serengeti) had become contaminated with the Muguga isolate. Unfortunately, since early passages of *T. parva* (Serengeti) were no longer available, it was not possible to determine when this contamination had occurred during its passage history. However, cross-challenge experiments with the Muguga and Serengeti isolates reported in 1973 showed that animals immunised with Muguga remained susceptible to challenge with Serengeti (Young et al., 1973), suggesting that the parasites were antigenically distinct at that time. A third instance of adaptation of a Kenyan buffalo isolate of *T. parva* to tick transmission between cattle was reported by Maritim and colleagues in 1992. Again, the levels of piroplasm parasitaemia and the efficiency of transmission progressively increased during five tick passages through cattle. Significantly in this study, examination of the parasites using a panel of monoclonal antibodies demonstrated that they retained a similar antibody profile during passage, including reactivity with an antibody that usually only reacts with buffalo-derived parasites. It is of note that one of the cloned populations of *T. parva* sporozoites subsequently produced by Morzaria and colleagues (1995) was generated from the same buffalo-derived isolate used in the experiments reported by Maritim et al. (1992). This cloned population was obtained by tick pick-up from an animal infected with a cloned derivative from a bovine cell line infected in vitro with the buffalo *T. parva* isolate. Although this clone is clearly tick transmissible, the level of infection produced in ticks is lower than that observed with clones of cattle-maintained *T. parva*.

In contrast to the findings with East African parasites, transmission of South African buffalo-derived *T. parva* from cattle has proved extremely difficult and there are no reports of “transformation” of the parasite behaviour. Until recently, despite numerous attempts, there was only one report of experimental transmission from a splenectomised calf (Neitz, 1957). However, in the last year Latif et al. (2019) reported successful experimental transmission following tick feeding on one of four cattle that had recovered from natural infection with buffalo-derived *T. parva* during a disease outbreak in the KwaZulu-Natal province of South Africa.

## 7 Antigenic heterogeneity in *T. parva*

Prior to the 1970s there was little information on antigenic heterogeneity among populations of *T. parva.* The prevailing view was that cattle that recovered from infection with cattle *T. parva* were immune to further challenge. However, in the course of studies conducted in the 1970s (Radley et al., 1975a, Radley et al., 1975b), which led to the development of an infection and treatment method of vaccination, it became apparent that cattle immunised with a single *T. parva* isolate were not protected against experimental challenge with some heterologous isolates or against field challenge. These studies were greatly aided by development of methods to produce cryopreserved stabilates of sporozoites (Cunningham et al., 1973), which allowed reproducible experimental studies with standardised doses of parasites of defined content. By using a mixture of three different parasite isolates for immunisation, Radley et al., 1975a, Radley et al., 1975b were able to obtain broad protection against experimental challenge with different cattle isolates (Radley et al., 1975b) and against field challenge with predominantly cattle-derived *T. parva* (Uilenberg et al., 1976). This mixture of three parasite isolates (referred to as the Muguga cocktail) has been used successfully to vaccinate cattle in the field (Di Giulio et al., 2009, Martins et al., 2010). However, Radley et al. (1979) showed that a proportion of cattle immunised with the Muguga cocktail were not protected against experimental challenge with several buffalo-derived parasite isolates and against challenge in a paddock that had been “cleaned” of potential infection and then seeded with buffalo-derived *T. parva* by introducing clean ticks and two infected buffalo (Young et al., 1977a). In an earlier study (Young et al., 1977b), involving introduction of cattle into the same paddock, cattle were immunised separately with three different isolates from the buffaloes used to seed the paddock; protection was observed with two of the isolates, which had been generated by pooling several tick feeds, whereas the third isolate derived from a single tick feed failed to give protection. These results indicated that the buffalo parasite population in the paddock differed antigenically from the parasites in the Muguga cocktail and provided evidence of antigenic heterogeneity among the buffalo-derived parasites. Two recent studies, in which groups of naïve or Muguga cocktail-vaccinated cattle were introduced into field sites grazed solely or predominantly by buffalo (Bishop et al., 2015, Sitt et al., 2015), revealed that the vaccinated cattle remained fully susceptible to natural challenge, clearly demonstrating antigenic disparity between buffalo-derived parasites and the parasites used in the vaccine.

The findings discussed above demonstrate clear differences in the ability of *T. parva* isolates to cross-protect. However, while the results collectively demonstrated that animals immunised with cattle *T. parva* often showed incomplete protection, invariably immunisation with buffalo-derived parasites protected against challenge with cattle *T. parva*.

## 8 Genotypic composition of *T. parva* populations

### 8.1 Early observations

A major drawback of early studies was the lack of laboratory reagents and methods to characterise different parasite populations. During the 1980s, development of parasite-specific monoclonal antibodies and DNA probes for the first time provided tools to examine parasite diversity. These studies confirmed antigenic and molecular differences between parasite isolates of both cattle and buffalo origin and, in some cases, between clonal components of the same isolate (Minami et al., 1983, Conrad et al., 1987a, Conrad et al., 1987b, Conrad et al., 1989; Bishop et al., 1989). Using DNA probes, the restriction fragment length polymorphism (RFLP) profiles obtained with buffalo isolates often differed substantially from those of cattle parasites and generally were more heterogeneous. Monoclonal antibodies and DNA probes also revealed parasite heterogeneity within buffalo cell lines and in ex vivo buffalo samples (Conrad et al., 1989, Bishop et al., 1993, Oura et al., 2011b); up to nine alleles of satellite markers were detected in an individual animal (Oura et al., 2011b). Nevertheless, the results overall did not provide a means of clearly distinguishing parasites derived from buffalo and cattle; nor did the identified differences correlate with findings on cross-protection. Moreover, since most of the monoclonal antibodies were specific for a single polymorphic parasite protein (the polymorphic immunodominant molecule – PIM) (Toye et al., 1991) and most studies employing DNA probes used in RFLP studies targeted a single repetitive DNA element (TpR), these tools provided limited resolution at the genome level. In the last 15 years, the development of genome sequencing and application of a number of new molecular typing methods opened up opportunities to interrogate the genetic composition of parasite populations in greater detail. The publication of the first genome sequence for *T. parva* in 2005 (Gardner et al., 2005) was particularly significant in this regard. At the same time, the identification of a series of antigens recognised by T cell-mediated immune responses considered to be important in immunity (Graham et al., 2006, Graham et al., 2008) has allowed immunologically relevant analyses of antigenic heterogeneity.

### 8.2 Genome sequencing

The full genome sequence of a clone of *T. parva* (Muguga) reported by Gardner et al. (2005) revealed a genome of 8.3 × 10^6^ bp, predicted to encode 4035 proteins. Sequences have subsequently been reported for the three parasite isolates used in the Muguga cocktail vaccine (Norling et al., 2015) and for a further nine isolates from various locations in East Africa, two of which were of buffalo origin (Hayashida et al., 2013). The cattle-derived *T. parva* genomes in the latter study contained between 34,814 and 51,790 single nucleotide polymorphisms (SNPs) compared with the reference Muguga sequence, whereas the two buffalo isolates had over twice this number of SNPs. These data suggest divergence of buffalo from cattle *T. parva*, but larger numbers of sequences are required to draw firm conclusions on this. More generally, the available sequence data have proved invaluable for identifying genetic markers and for refining strategies for antigen identification.

### 8.3 Infections are genotypically mixed, facilitating sexual recombination

The use of satellite DNA markers identified from the parasite genome sequence demonstrated that different parasite isolates had unique molecular fingerprints. Typing of carrier cattle in the field with these reagents revealed that most infections were genotypically mixed and that the genetic complexity of the parasites in the blood of infected animals increased with the age of the animals (Oura et al., 2003, Oura et al., 2005, Oura et al., 2011a, Oura et al., 2011b). This finding implied that cattle acquire multiple infections over time and was consistent with experimental findings that immunity against *T. parva,* which protects against clinical disease, does not prevent establishment of infection upon parasite challenge (eg. Radley et al., 1975b). Analyses of the genotypes of the parasite populations in local cattle populations have indicated that although there is some geographical sub-structuring of parasite genotypes (Oura et al., 2005, Silih et al., 2018), overall the populations appear panmictic, suggesting frequent genetic exchange.

Experimental studies confirmed the capacity of *T. parva* to undergo sexual recombination by examining the genotypes of parasites from ticks that had fed on animals co-infected with two genotypically distinct clones of *T. parva* (Henson et al., 2012, Katzer et al., 2011). These results, coupled with the observation that ticks in the field frequently feed on animals infected with mixed genotypes, further support the view that sexual recombination is a frequent event in *T. parva*.

### 8.4 Buffalo *T. parva* populations exhibit greater genetic diversity

Detailed analyses of the immune responses of cattle to *T. parva* have shown that immune cattle generate strong T cell responses against infected leukocytes (reviewed in Morrison et al., 2015) and have provided compelling evidence that CD8 T cell responses play a key role in immunity (McKeever et al., 1994, Taracha et al., 1995). This work led to the identification of a number of antigens recognised by CD8 T cells from immune cattle (Graham et al., 2006) and detailed studies of two of these antigens (Tp1 and Tp2) demonstrated polymorphism that resulted in differential recognition by CD8 T cells (MacHugh et al., 2009, Connelley et al., 2011). Using the genes encoding these antigens as genetic markers, Pelle et al. (2011) undertook an analysis of sequence polymorphism in a series of 79 cell lines infected with *T. parva.* They examined cell lines that had either been infected in vitro with laboratory-maintained strains of *T. parva* or were isolated from naturally infected cattle or buffalo in various field locations. The cattle isolates included parasites from cattle in buffalo-free areas or from sentinel cattle introduced into an area populated predominantly by buffalo. The Tp1 and Tp2 genes proved to be highly polymorphic, showing SNPs in 12% and 61% of residues, resulting in 30 and 41 protein variants, respectively, among the 79 isolates examined. However, the most striking finding was that the isolates from buffalo or from cattle exposed to buffalo-grazed pasture displayed much greater sequence diversity compared with those from cattle in buffalo-free areas. Thus, only nine of the 30 Tp1 amino acid sequence variants and three of the 41 Tp2 variants were present in the cattle-maintained parasite population. However, the majority of these cattle variants were also found among the buffalo and buffalo-associated populations, indicating that they do not represent a completely divergent population. Based on these findings, the authors concluded that the cattle-maintained parasites represent a subset of the *T. parva* population and, coupled with other evidence, suggested that these parasites are most likely to have been selected for their ability to undergo tick transmission between cattle.

Further evidence of extensive heterogeneity in buffalo *T. parva* was obtained in a study that performed high-throughput sequencing of PCR amplicons of segments of six antigen-encoding genes (including Tp1 and Tp2) using DNA obtained directly from buffalo blood samples (Hemmink et al., 2018). In addition to confirming a high level of allelic diversity of each gene at the population level, the results revealed extensive allelic diversity within individual buffalo. For example, between 11 and 24 nucleotide sequence variants were detected for each of the six genes in one buffalo sample. Given that the infection rate of ticks with *T. parva* in the field is usually <2% and that many infected ticks have only one or two infected salivary gland cells (each derived from a single *T. parva* zygote), these results clearly indicate that the parasites in these buffalo must have arisen from multiple infection events. The simultaneous presence of multiple parasite genotypes in buffalo blood again highlights the enormous potential for genetic recombination to occur in ticks feeding on infected buffalo.

The same study included a comparison of sequences obtained from buffalo in Kenya and South Africa. Samples from both regions showed similar levels of sequence diversity both at the population level and within animals. Although there was evidence for differentiation of the Kenyan and South African sequences, analyses of molecular variance for each gene revealed that the majority of the underlying nucleotide sequence polymorphism was common to both areas. Moreover, comparison of the sequences of known CD8 T cell epitopes within the two most polymorphic genes, Tp1 and Tp2, revealed a high level of similarity in epitope variants in the Kenyan and South African samples, including epitope sequences in the South African samples that were identical to those found in East African cattle-maintained *T. parva*. These findings indicated that much of the sequence variation in these genes pre-dates geographical separation of the parasites.

The p67 *T. parva* sporozoite surface protein is involved in initial binding of sporozoites to bovine lymphocytes leading to cell invasion (Dobbelaere et al., 1985, Nene et al., 1992). Certain monoclonal antibodies specific for p67 neutralise the infectivity of sporozoites for lymphocytes in vitro (Dobbelaere et al., 1984, Musoke et al., 1984)*.* Moreover, immunisation of cattle with recombinant p67 incorporated in adjuvant has been shown to protect a proportion of animals against parasite challenge (Musoke et al., 1992). To date, no variation in the sequence of the gene encoding p67 has been found in cattle-maintained *T. parva*. However, a sequence variant, which differed by the presence of an insert of 43 amino acids, was detected in an East African buffalo-derived parasite (Nene et al., 1996). A subsequent study in South Africa, which examined the sequences of p67 in buffalo-derived isolates, identified a further two sequence variants, in addition to the cattle and buffalo sequences reported in East Africa (Sibeko et al., 2010). The observed polymorphism affected sites of antibody epitopes within the protein (Obara et al., 2015), although the effect on recognition by the respective monoclonal antibodies was not examined. The possibility that the sequence of p67 influences the ability of buffalo-derived parasites to infect cattle was addressed in a study that examined p67 sequences of parasites infecting cattle introduced into an area previously only grazed by buffalo, compared with sequences from buffalo in the same location (Sitt et al., 2019). Over 40 sequence variants of p67 were identified, most representing minor variants of the previously described alleles; many of the variants were found in both cattle and buffalo and there was no significant difference in the level of sequence diversity in the two host species. Up to five different sequence variants were found in individual cattle, indicating multiplicity of infection. These findings failed to reveal any restriction in the ability of buffalo parasites expressing different alleles of p67 to infect cattle, and added to the evidence that *T. parva* transmitted to cattle from buffalo are much more genotypically diverse than cattle-maintained *T. parva*.

The similar diversity seen in buffalo and cattle *T. parva* in this study appears inconsistent with an earlier study in Uganda, which observed distinct satellite DNA marker profiles in parasites from cattle and buffalo grazing in close proximity (Oura et al., 2011a, Oura et al., 2011b). However, this almost certainly is due to the latter study having examined animals with carrier infections rather than the acute infections studied by Sitt et al. (2019); as discussed above, such carrier infections are likely to originate only from challenge with cattle-maintained *T. parva*.

## 9 Practical implications for disease control

Due to the acute onset and fatal nature of disease caused by *T. parva* in cattle, control measures focus predominantly on disease prevention. In eastern Africa, dipping or spraying cattle with acaricides is widely practiced, and in some areas vaccination of cattle by the “infection and treatment” method is used. The shortcomings of these control methods and efforts to develop alternative, more sustainable methods of vaccination have been reviewed elsewhere (Morrison and McKeever, 2006, Nene and Morrison, 2016). With regard to vaccination, antigenic diversity in *T. parva* is a key issue, whether using the current vaccine or considering future alternatives.

Despite the ability of the Muguga cocktail vaccine to protect cattle against field challenge, predominantly with cattle-maintained *T. parva*, we have found that it contains relatively limited genotypic and antigenic diversity (Hemmink et al., 2016). The protective CD8 T cell response to *T. parva* exhibits profound immunodominance, whereby responses induced by single *T. parva* isolates in individual animals are focused on a few dominant polymorphic antigens (MacHugh et al., 2009). We have proposed that, in addition to polymorphism of the CD8 T cell antigens, this immunodominance is a key determinant of strain specificity (Morrison et al., 2015). Use of several parasite isolates (as in the Muguga cocktail), each of which induces an independent immune response, is expected to result in responses to a wider range of antigens. Moreover, further broadening of the antigenic specificity of the response is likely to occur following field challenge of vaccinated animals. Due to the panmictic nature of the *T. parva* population, such responses will allow recognition of most cattle-derived challenge parasites, even though the vaccine does not incorporate all of the antigenic diversity found in the challenge population.

The data discussed in previous sections of this review have highlighted the extent of antigenic diversity in the buffalo *T. parva* population and the failure of the Muguga cocktail infection and treatment vaccine to provide protection against buffalo challenge. Despite this diversity, vaccination with the Muguga cocktail has been effective in some areas where buffalo are present (Di Giulio et al., 2009, Martins et al., 2010). As argued elsewhere (Morrison et al., 2015), this may be due to a relatively low abundance of buffalo-fed versus cattle-fed ticks in these areas. However, vaccine-induced immunity is likely to be breached in some situations where cattle are farmed alongside a high density of wildlife or in close proximity to game reserves with buffalo populations. More information on the *T. parva* populations carried by ticks in such situations is required to provide a better understanding of the nature of the parasite challenge in relation to the risk of vaccine breakthrough.

Further improvement of vaccination by infection and treatment by incorporation of buffalo-derived parasites is problematic due to poor tick transmissibility of most buffalo isolates in cattle, thus hampering production of parasites for vaccination. In addition, the enormous diversity among the buffalo parasites may hinder the selection of representative buffalo-derived isolates for inclusion in a vaccine. Hence, vaccination against these parasites may need to await development of alternative vaccines.

In South Africa, the disease threat is confined to challenge with buffalo-derived parasites and the control strategy for many decades has been to prevent contact between infected buffalo and cattle by fencing and restriction of animal movement (Potgieter et al., 1988, Lawrence, 1990). Detailed surveillance of livestock is conducted to ensure rapid identification of outbreaks in cattle and measures are imposed on infected farms to prevent spread of infection. In the last few decades, this situation has become complicated by expansion of game ranching for commercial hunting of wildlife, which has involved sales and movements of game species between ranches (Michel and Bengis, 2012). Testing of buffalo for *T. parva* prior to movement is required to avoid spread of infection. Although we have argued above that the likelihood of buffalo *T. parva* adapting to transmission and maintenance in cattle seems remote in South Africa, the potentially disastrous consequences of such a rare event fully justifies the control measures currently being applied.

## 10 Unanswered questions

### 10.1 When did infection of buffalo first occur in South Africa?

One of the curious aspects of the historical data is the apparent absence of buffalo-derived *T. parva* from South Africa prior to its recognition in the 1950s. One suggested explanation (Norval et al., 1992) is that infection of buffalo might have originated from transmission of infection to buffalo (and presumably gradual establishment and spread in the buffalo population) of parasites introduced by the imported cattle that gave rise to the initial outbreak of ECF in southern Africa. However, these cattle almost certainly did not carry buffalo-adapted parasites and given the uncertain ability of cattle *T. parva* to establish persistent infection in buffalo (see Section 10.2 below), this suggestion seems untenable. It is also inconsistent with the extensive genotypic diversity observed in the current South African buffalo parasites. The similar composition of these parasites to those in buffalo found further north suggests that they have arisen from southward movement of buffalo or buffalo-to-buffalo transmission of their parasites. This might have been due to changes in climatic conditions in the region. An alternative suggestion put forward by Lawrence et al. (1994b) for Zimbabwe is that the parasite might have been present in buffalo prior to recognition of the disease in cattle, but was masked by the continued presence of ECF and the focus of the authorities on eradicating ECF.

### 10.2 Why has genotypic restriction of *T. parva* in cattle been maintained?

Given the assertion that *T. parva* in cattle was originally established by selection of parasites with the ability to undergo tick transmission between cattle, repeated selection might have been expected to result in greater diversity in the cattle parasites. In particular, if cattle-adapted parasites were transmitted to buffalo, recombination with buffalo parasites in the course of tick passage could potentially have resulted in genotypically diverse parasites that are transmissible back to cattle. Only a few old experimental studies have investigated infection of buffalo with cattle *T. parva* (Lewis, 1943, Barnett and Brocklesby, 1966b) and these yielded variable results including successful transmission from occasional animals. However, given the inconsistency of the results and the uncertain infection status of the animals used in the experiments, further studies are needed to determine whether cattle *T. parva* can establish persistent, tick-transmissible infection in buffalo. Indeed, it remains possible that adaptation of *T. parva* to cattle has resulted in parasites that are poorly adapted to infection and transmission in buffalo.

The demonstration that experimental tick transmission of buffalo parasites in cattle can sometimes result in selection of parasites adapted to transmission between cattle would imply that such adaptation might continue to occur in the field. However, these studies relied on several passages of the same parasite with ticks fed on cattle during the acute phase of infection and applied in large numbers to further naïve cattle. In an endemically infected field situation, where such parasites are likely to be transmitted together with cattle-adapted parasites, the capacity for selection would be greatly reduced and they may readily be out-competed by the highly transmissible cattle parasites. Moreover, the capacity of the selected buffalo parasites to be maintained in cattle may well depend on their ability to establish a carrier state. While two of the reported transformed populations of *T. parva* were found to establish a carrier state (Barnett and Brocklesby, 1966a, Young and Purnell, 1973), it is unclear whether the earlier passages of these parasites had this capability. Theoretically, there might be greater capacity to select cattle-transmissible parasites in South Africa, where the cattle are fully susceptible to infection and are not exposed to cattle-maintained *T. parva*. However, the seasonal nature of parasite transmission renders this unlikely because the parasites would need to rapidly acquire the ability to establish a carrier state in order to survive beyond the first season of tick transmission.

### 10.3 Do similar population structures prevail in areas co-grazed by cattle and buffalo?

While genotyping studies of cattle-derived *T. parva* have examined parasites from diverse locations, these have been predominantly from areas with no or few buffalo. There is therefore a need to extend these analyses to include parasites from areas where cattle are farmed in the presence of a sizable buffalo population, to determine whether additional genotypes are identifiable among the cattle-maintained parasites.

### 10.4 What is the mechanism by which buffalo *T. parva* adapt to transmission in cattle?

Despite the evidence that repeated tick passage of some buffalo-derived *T. parva* through cattle can result in “transformation” of the behaviour of the parasite to resemble that of cattle-maintained *T. parva*, many other attempts to achieve tick passage of buffalo isolates through cattle (eg. Schreuder et al., 1977), including most attempts with South African isolates, have failed. The absence of detectable piroplasms in most cattle infected naturally or experimentally with buffalo *T. parva* indicates an inability of these parasites to differentiate to the tick-infective piroplasm stage in cattle. Those parasites that adapt to experimental passage in cattle must have had some residual capacity to differentiate to the piroplasm stage in cattle. Indeed, in one of the reports of parasite transformation (Maritim et al., 1989), most of the cattle used for the first passage had low levels of microscopically detectable piroplasms, although in other reports piroplasms were usually not detected until the second or third passage.

The mechanism by which buffalo parasites undergo “transformation” during passage in cattle is unknown. In recent years, epigenetic mechanisms of regulating gene expression that affect cellular phenotypes have received increasing attention (Lowdon et al., 2016). Studies of *Plasmodium* parasites have implicated epigenetic processes in regulating a number of biological activities including expression of variable antigen genes and differentiation of the sexual stages of the parasites in erythrocytes (reviewed in Cortes and Deitsch, 2017). However, to date these findings have only been documented across generations of asexually replicating parasites, and there is as yet no evidence of inheritance of epigenetically determined traits following completion of the sexual cycle of the parasite.

Given the evidence of extensive genotypic diversity of *T. parva* in infected buffalo (Hemmink et al., 2018), it is clearly plausible that “transformation” of the parasite following tick passage is the result of genetic selection from within mixed populations. The three documented reports of “transformation” used multiple cattle, not all of which became infected, and only some passage lineages yielded transformed parasites, suggesting heterogeneity within the parasite isolate used and possible selection upon passage. A study reported by Maritim et al. (1989) also provided evidence of antigenic selection following a single tick passage of a buffalo *T. parva* isolate in cattle. When cattle were immunised with buffalo-derived parasites isolated after cattle passage, most animals were not protected against challenge with the parent buffalo isolate, indicating loss of antigenic diversity. Transformation from the buffalo to cattle phenotype is unlikely to be due to a single gene, otherwise selection of adapted parasites would occur rapidly due to vastly superior transmissibility of the “transformed” parasites. Rather, multiple genes are likely to be involved, in which case the fully cattle-adapted genotypes are unlikely to pre-exist in the starting buffalo parasite population but may require recombination during the tick passages to generate the appropriate genotype. Molecular tools and methods are now available to address these questions.

Given the above model for a genetic basis of selection of transformed parasites, the selection of such parasites under field conditions is much less likely. Unlike the experimental system that maximises co-transmission of multiple parasite genotypes at each passage by using large numbers of ticks, any ticks that pick up parasites from a naturally infected animal will be rapidly diluted by feeding on different hosts at the next developmental stage.

In practical terms, elucidation of a genetic basis of transmissibility in cattle would provide markers to distinguish cattle- and buffalo-derived parasites in areas co-grazed by both species and to identify buffalo parasites that could potentially adapt to transmission in cattle.

### 10.5 Are similar host relationships observed for other Theileria spp.?

A number of other *Theileria* spp. are also able to infect non-definitive hosts, but infection is not maintained in these hosts. These include *Theileria sp.* (buffalo) (Bishop et al., 2015) and *Theileria taurotragi* (Grootenhuis et al., 1979), which are parasites of African buffalo and eland, respectively, both closely related to *T. parva*. They are able to infect cattle and, in the case of *T. taurotragi*, there is experimental evidence of tick transmission of infection from cattle during the acute phase of infection. However, there is no evidence that either parasite establishes persistent infection or that infection can be maintained in cattle. The cattle parasite *T. annulata* has been shown to infect sheep, both naturally and experimentally, but (as with buffalo-derived *T. parva* in cattle) *T. annulata* does not differentiate to the tick-infective piroplasm stage in sheep (Leemans et al., 1999a, Leemans et al., 1999b). In this regard, it has been suggested that the closely related pathogenic sheep parasite *Theileria lestoquardi* may have evolved by adaptation of an ancestral *T. annulata* to transmission in sheep (Al-Hamidhi et al., 2016). These examples indicate that the ability to undergo differentiation to tick-infective stages and to persist beyond the acute phase of infection are key parameters in determining the host tropism of *Theileria* parasites.

### 10.6 Is the designation of cattle and buffalo *T. parva* as a single species justified?

For eukaryotic pathogens that undergo sexual recombination, the ability of genotypically different strains to recombine is one of the parameters that defines whether they belong to the same species. While the genetic and antigenic similarity of *T. parva* in cattle and buffalo suggests that they are likely to be able to undergo recombination, this has not been demonstrated directly and it remains possible that recombination of cattle-maintained *T. parva* with some of the more divergent buffalo parasites is incompatible. In any event, the evidence that the parasites appear to be maintained largely as separate populations in the two hosts means that they have little, if any, opportunity to undergo recombination. Hence, the issue of species definition must remain an open question, which requires further, more comprehensive, data to resolve.
